# Metagenome and Metatranscriptome Revealed a Highly Active and Intensive Sulfur Cycle in an Oil-Immersed Hydrothermal Chimney in Guaymas Basin

**DOI:** 10.3389/fmicb.2015.01236

**Published:** 2015-11-10

**Authors:** Ying He, Xiaoyuan Feng, Jing Fang, Yu Zhang, Xiang Xiao

**Affiliations:** ^1^State Key Laboratory of Microbial Metabolism, School of Life Sciences and Biotechnology, Shanghai Jiao Tong UniversityShanghai, China; ^2^State Key Laboratory of Ocean Engineering, Shanghai Jiao Tong UniversityShanghai, China; ^3^Institute of Oceanology, Shanghai Jiao Tong UniversityShanghai, China

**Keywords:** hydrothermal vent, metagenomics, metatranscriptomics, sulfur cycle, carbon cycle

## Abstract

The hydrothermal vent system is a typical chemosynthetic ecosystem in which microorganisms play essential roles in the geobiochemical cycling. Although it has been well-recognized that the inorganic sulfur compounds are abundant and actively converted through chemosynthetic pathways, the sulfur budget in a hydrothermal vent is poorly characterized due to the complexity of microbial sulfur cycling resulting from the numerous parties involved in the processes. In this study, we performed an integrated metagenomic and metatranscriptomic analysis on a chimney sample from Guaymas Basin to achieve a comprehensive study of each sulfur metabolic pathway and its hosting microorganisms and constructed the microbial sulfur cycle that occurs in the site. Our results clearly illustrated the stratified sulfur oxidation and sulfate reduction at the chimney wall. Besides, sulfur metabolizing is closely interacting with carbon cycles, especially the hydrocarbon degradation process in Guaymas Basin. This work supports that the internal sulfur cycling is intensive and the net sulfur budget is low in the hydrothermal ecosystem.

## Introduction

Hydrothermal vents are often discovered in ocean ridges where hydrothermal fluid is emitted after the hydrothermal circulation and alteration of seawater entrained through geothermally heated subseafloor basalt (Von Damm, 1990). The deep-sea hydrothermal vent fluid is commonly characterized by its high temperature, varied salinity, enriched metallic elements, and particularly high contents of reduced chemicals, such as H_2_, CH_4_, and H_2_S (Jannasch and Mottl, 1985). A thermodynamic non-equilibrium is created when the hydrothermal vent fluid encounters sea water that is cold and at a rather high oxidative state, which allows various abiotic and biotic reactions occur. Thus, the hydrothermal vent system is a typical chemosynthetic ecosystem in which microorganisms play essential roles in the generation, consumption, and modification of energy available in the environment (Reysenbach and Shock, 2002).

In the hydrothermal vent ecosystem, almost all types of inorganic sulfur compounds (e.g., S^2-^, S, S_2_O_2_^2-^, SO_2_, S_2_O_3_^2-^, and SO_4_^2-^) are abundant and actively converted through chemosynthetic pathways to provide energy and thus sustain the microbial population in the ecosystem (Nakagawa et al., 2005). For example, in the Lost City hydrothermal field, the dominant *Thiomicrospira*-like group, which consists of sulfur-oxidizing chemolithoautotrophs, was observed in the carbonate chimney (Brazelton and Baross, 2010). In the Lau Basin hydrothermal vent field, sulfur-oxidizing Alphaproteobacteria, Gammaproteobacteria, and Epsilonproteobacteria have been suggested to be dominant in the exterior chimney, whereas putative sulfur-reducing Deltaproteobacteria are dominant in the interior of the chimney (Sylvan et al., 2013). In the Guaymas Basin hydrothermal vent field, sulfate-reducing microorganisms, e.g., Desulfobacterales, have been detected and are hypothesized to be involved in the anaerobic methane-oxidation process (Biddle et al., 2012). Moreover, the sulfur cycling is alternated by the chemical reactions that occur during the emitting and growth of the hydrothermal vent. Reduced sulfur compounds are extremely sensitive to oxidants and easily precipitated with metal ions to form chimney or nodule structures (Orcutt et al., 2011). Moreover, shifts in temperature and fluid composition have been observed during the life span of a hydrothermal vent. For example, at 9°N East Pacific Rise, Bio9 vent fluids were 368°C in 1991, increased to an estimated temperature greater than or equal to 388°C after a second volcanic event in 1992, and thereafter declined over the next similar to 2 years reaching a temperature of 365°C in December 1993 (Fornari et al., 1998). The hydrogen concentration in the hydrothermal plum in the NE Lau Basin dropped from 14843 nM in 2008 to 4410 nM in 2010 then further to 7 nM in 2012 (Baumberger et al., 2014). As a result, environmental fluctuations may be induced between sulfate- and sulfur-reducing archaea and contribute to the diverse roles of these microorganisms in the ecosystem (Teske et al., 2014). Therefore, a better understanding of sulfur cycling is essential for describing the geobiochemistry and providing hints to identify the life status of a hydrothermal vent ecosystem.

Due to the complexity of microbial sulfur cycling resulting from the numerous parties involved in the process, the sulfur budget in a hydrothermal vent is poorly characterized. To date, most studies have focused on the abundance and diversity of sulfur oxidizers and sulfate reducers in environmental samples through a metagenomic approach (Nakagawa et al., 2005). The exception is the study conducted by Anantharaman et al. (2013), who combined metatranscriptomic and metagenomic analyses of a hydrothermal plume sample and demonstrated the novel metabolic potentials of the SUP05 group of uncultured sulfur-oxidizing Gammaproteobacteria. However, this finding is based on the near-complete genomes of two SUP05 populations, and the information is restricted to this particular group of sulfur oxidizers (Anantharaman et al., 2013). The in-depth mining of the metatranscriptomic data remains too scarce to allow construction of the entire sulfur cycle and thus further illustrate the interactions of this process with the biological cycling of C, N, and O elements.

The Guaymas Basin in the Gulf of California is a young marginal rift basin characterized by the active hot venting of reduced sulfur compounds and the rapid deposition of organic-rich sediments. These features make the sulfur cycle in this ecosystem particularly intensive and closely interact with the carbon cycle, including hydrocarbon degradation (Bergmann et al., 2011). Thus, this sampling site is ideal for illustrating all of the possible microbial sulfur metabolic pathways and to evaluate the maximal biomass contribution of sulfur-metabolizing microorganism to the hydrothermal vent ecosystem. In this study, we performed an integrated metagenomic and metatranscriptomic analysis on a chimney sample from Guaymas Basin to achieve a comprehensive study of each sulfur metabolic pathway and its hosting microorganisms and constructed the microbial sulfur cycle that occurs in the site.

## Results

### Composition of the Microbial Community

**FIGURE 1:**
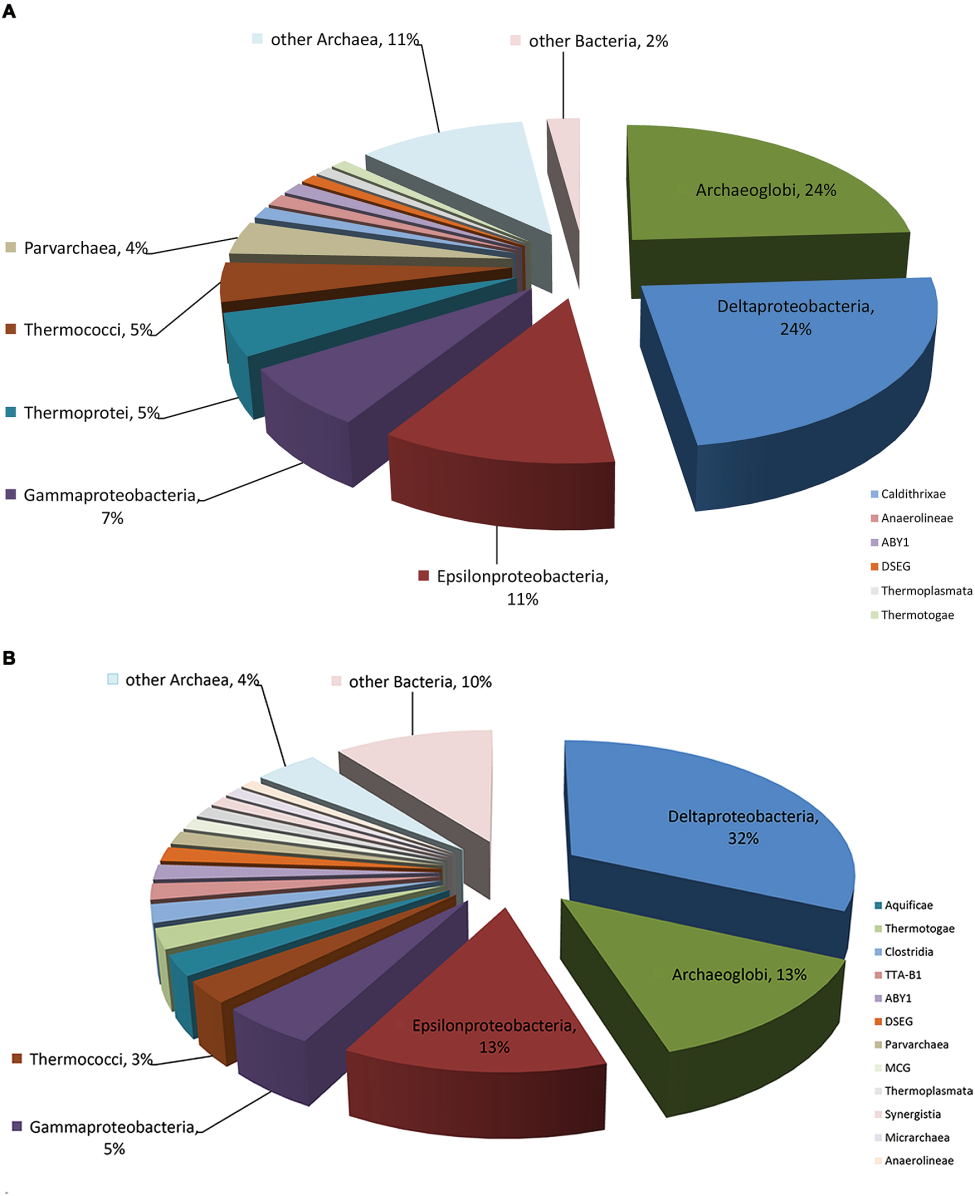
**Microbial composition of the enriched AOM-SR community.** Detailed information is displayed in **Table 1**. **(A)** Percentage of the microbial community determined from the 16S rRNA gene sequences retrieved from the metagenome. **(B)** Percentage of the microbial community determined from the 16S rRNA gene sequences retrieved from the metatranscriptome.

The composition and function of this microbial community were assessed at both the DNA and RNA levels to estimate the community metabolic potential and activity, respectively. The metagenome and metatranscriptome sequencing resulted in 199,903,215 and 1,885,022,958 bp clean sequences, respectively (**Table 1**). The metagenome raw reads were assembled into 49,055 contigs with an average length of 544 bp. In total, 5,417,253 reads (26.2%) from the metatranscriptome were mapped onto metagenomic contigs for quantification of the gene transcripts. 222 and 690,059 16S rRNA gene fragments were identified from the metagenome and metatranscriptome, respectively. The class-level taxonomic compositions of the metagenome and metatranscriptome revealed obvious differences in the presence and the activity of microbes in this community (**Table 1**). At the DNA level (**Figure 1A**), Archaeoglobi were found to be the most abundant, with 24.0% of the sequences assigned, and followed by Deltaproteobacteria (23.6%) and Epsilonproteobacteria (11.3%). At the RNA level (**Figure 1B**), the same dominant groups were found: Deltaproteobacteria (31.8%), Archaeoglobi (13.3%), and Epsilonproteobacteria (12.8%). As reported previously (He et al., 2013), 53,034 gene features were predicted and then followed by manual examination and 19,491 gene features (36.8%) were considered to have expressions determined by transcriptomic reads mapping (see Materials and Methods). A total of 8929 (45.3%) and 4628 (23.7%) of all of the expressed genes were assigned (based on the BLAST results as described in Section “Materials and Methods”) to Bacteria and Archaea, respectively, and the remaining sequences were not assigned to any category. Among the 13,557 expressed genes with taxonomic information, 2135 (15.7%) were from the highly abundant Archaeoglobi, which is consistent with the results from the 16S rRNA gene analysis. Although the assignment of bacterial genes could not be resolved well at the family level, the dominance of Deltaproteobacteria and Epsilonproteobacteria was still observed. As the archaeal cells typically have fewer copies of the 16S rRNA gene compared with bacterial cells, the proportion of active Archaeoglobi in this community was underestimated. Nevertheless, the predominant active players in this microbial community were Deltaproteobacteria, Archaeoglobi, and Epsilonproteobacteria.

**Table 1:** Summary of the metagenome and metatranscriptome.

	Metagenome	Metatranscriptome
Size of raw reads (bp)	199,903,215	1,885,022,958
Total no. of raw reads	512,830	20,714,538
Size of assembled contigs (bp)	26,703,275	–
Total assembled contigs	49,055	–
Average contig length (bp)	544	–
Average GC content of assembled contigs (%)	43	
Total no. of genes encoding in the contigs	53,034	–
Total no. of metatranscriptomic reads mapped to the metagenome	–	5,417,253
Total no. of 16S rRNA sequences	222	690,059


The *de novo* assembly of metagenomic reads and binning by tetranucleotide signatures (Dick et al., 2009) identified three genomic bins (**Supplementary Figure S1** and **Supplementary Table S1**). These three bins (herewith denoted bin20, bin21, and bin22) were assigned based on their phylogenomic marker genes to *Desulfobacteraceae*, Desulfovibrionales and *Archaeoglobus*. The identified genes in the obtained bins ranged from 486 to 1224. The genome completeness was estimated to range from ∼10 to 34%, based on single-copy gene estimation (**Supplementary Table S1**). These three genomic bins will improve the taxonomic assignment of the expressed genes and the reconstruction of the metabolic pathways.

### Sulfur Metabolism

**FIGURE 2:**
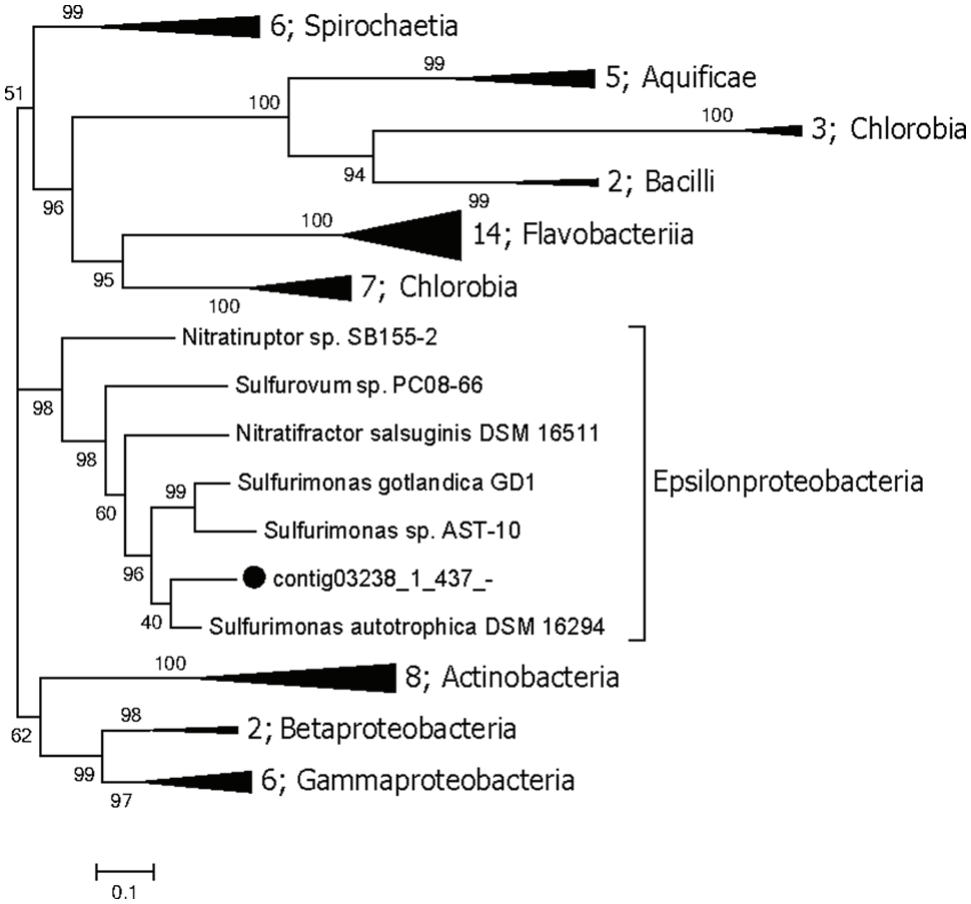
**Maximum likelihood phylogenetic tree of the *sqr* genes.** The bootstrap values are based on 1000 replicates, and the percentages are shown at the nodes. The genes identified in this study are highlighted with black dots. Numbers of genomes in each collapsed clade are displayed before the clade name.

**FIGURE 3:**
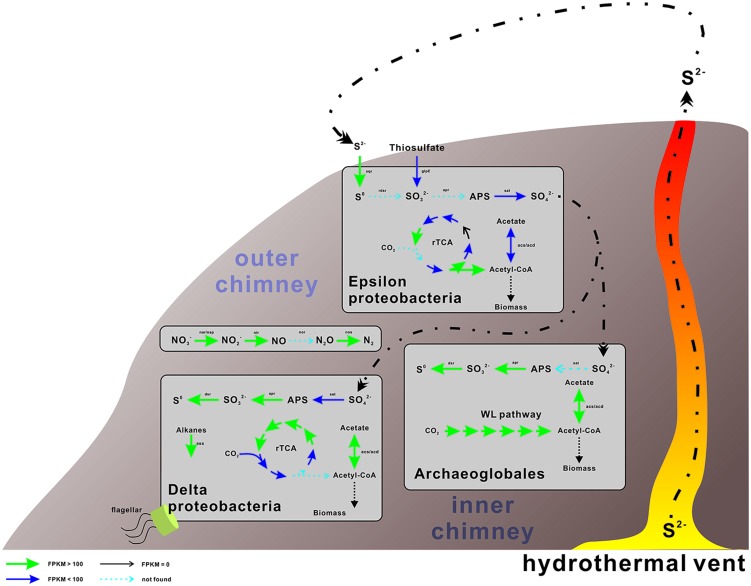
**Proposed metabolic and active pathways in this chimney community.** The expression and presence of the genes are indicated by the color and thickness of the arrows, as shown in the bottom-right side bar. For genes with multiple hits, only the genes with the highest expression value (FPKM, expected fragments per kilobase of transcript per million fragments mapped) are displayed and discussed. The processes conducted by Archaea are shown on the left, whereas those conducted by bacteria are presented on the right. Detailed information of these genes is displayed in **Tables 2**–**4**.

The genes involved in the oxidation of reduced sulfur (ORS) are sulfide quinone oxidoreductase (*sqr*), which mediates the oxidation of sulfide (HS^-^) to elemental sulfur (S^0^), the Sox enzyme complex (*soxABXYZ*), which is responsible for the oxidation of thiosulfate (S_2_O_3_^2-^) to elemental sulfur, the reverse dissimilatory sulfite reductase complex (*rdsr*), which is responsible for the oxidation of elemental sulfur to sulfite (SO_3_^2-^), and adenosine 5′-phosphosulfate reductase (*apr*) and sulfate adenylyltransferase (*sat*) for oxidation of sulfite to sulfate (SO_4_^2-^; Anantharaman et al., 2013). Conversely, the genes associated with the dissimilatory sulfate reduction (DSR) pathway (Fritz et al., 2002) are *sat*, *apr*, and sulfite reductase (*dsr*). The repertoire of genes associated with the ORS and DSR pathways were found to be expressed in this community (**Table 2**). Both *apr* and *dsr* were found at high expression levels in bin21 and bin22, confirming their active presence in SRB and *Archaeoglobus*. The *sqr* gene, key gene in the ORS pathway, is found present and active in Epsilonproteobacteria, of which the most highly expressed representative was classified into *Sulfurimonas* (**Figure 2**) that is one of the most abundant sulfur-oxidizing bacteria found in hydrothermal vent chimneys (Cao et al., 2014). The *sox* genes were not identified in either the metagenome or metatranscriptome (**Table 2**). In Epsilonproteobacteria, the proposed microorganism in the present study to perform the ORS pathway, *sat* gene was found to exhibit high and medium expression levels (**Table 2**). However, either *aprAB* or *dsrAB* was identified in the metagenome or metatranscriptome. This finding may be due to the fact that the 454-based metagenomes are still with low coverage and unable to present all the important functional genes. In Deltaproteobacteria and Archaeoglobales, which were proposed to conduct the DSR pathway in this study, *aprAB* and *dsrA* genes were found to be highly expressed in both of these two taxonomic groups, whereas *sat* and *dsrB* genes were found only in Deltaproteobacteria. The phylogenies of *aprA* and *dsrA* further confirmed their assignment to Deltaproteobacteria (**Supplementary Figures S2A,B**). In a previous study, the *aprA* with the highest abundance was assigned to the genus *Desulfobulbus* (Cao et al., 2014). In our study, the *aprA* gene with the highest expression was assigned to *Desulfovibrio* (**Supplementary Figure S2A**). To summarize, the taxonomic assignment and expression of key genes in the sulfur cycle suggest that both the ORS and DSR pathways are highly active in this oil-immersed microbial community, and the energy generated by the sulfur metabolism supports the dominant and active group (**Figure 3**).

**Table 2:** Genes identified in the sulfur metabolic pathway in the microbial community.

Gene name	Abbrevations	Deltaproteobacteria			Archaeoglobales			Epsilonproteobacteria	
				
		Assigned taxonomy^∗^		FPKM	Assigned taxonomy^∗^		FPKM	Assigned taxonomy^∗^		FPKM
							
		Bin	BLAST		Bin	BLAST		Bin	BLAST
Sulfate adenylyltransferase	*Sat*	–	Deltaproteobacteria	42.94	–	–	–	–	Epsilonproteobacteria	2.86
Adenylyl-sulfate reductase, subunit A	*aprA*	bin21	Desulfovibrionales	2502.51	bin22	*Archaeoglobus fulgidus*	761.68	–	–	–
Adenylyl-sulfate reductase, subunit B	*aprB*	bin21	Desulfovibrionales	235.17	–	*Archaeoglobus*	53.53	–	–	–
Sulfite reductase alpha subunit	*dsrA*	bin21	Deltaproteobacteria	221.81	bin22	*Archaeoglobus*	1914.15	–	–	–
Sulfite reductase beta subunit	*dsrB*	bin21	Deltaproteobacteria	1621.06			–	–	–	–
Sulfide:quinone reductase	*Sqr*	–	–	–	–	–	–	–	Epsilonproteobacteria	137.30


*The taxonomy assignments were determined by two methods, as described in Section Materials and Methods.” The binning index is explained in **Supplementary Table S1**. is based on the maximal expression value of the annotated genes.*

**FIGURE 4:**
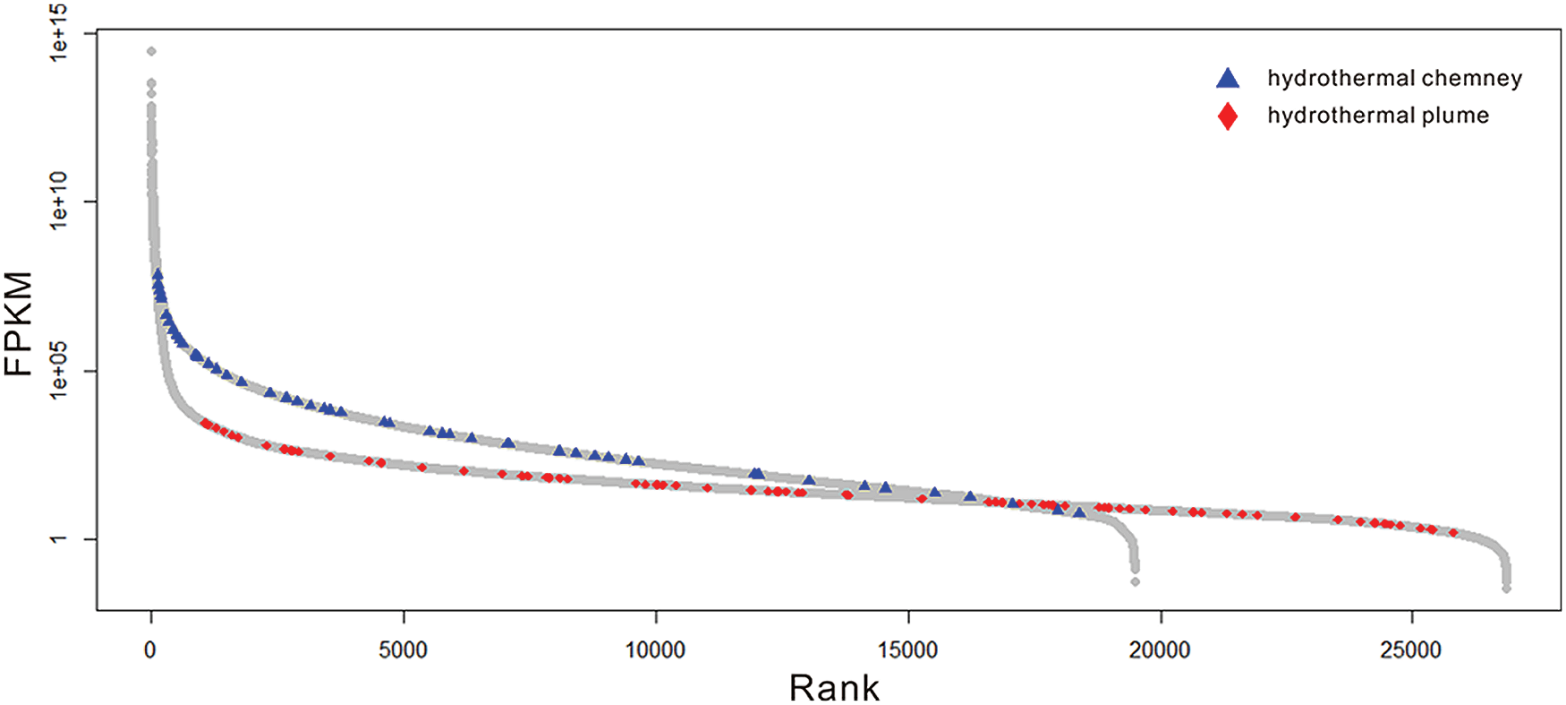
**Expression profiles of the genes in the metatranscriptome.** The gray circles indicate the genes of the whole community. The blue triangles and red diamonds represent those related to sulfur oxidation and reduction in this metatranscriptome and a previous study (Lesniewski et al., 2012), respectively. The relative abundance of the gene transcripts was normalized to the length of the gene fragment and the total number of all of the transcripts.

Because there are no metatranscriptome published for any hydrothermal vent chimneys, we compared the expression patterns of the sulfur-metabolizing genes in this metatranscriptome to those in the available metatranscriptome of a plum sample that was also collected from Guaymas Basin (Lesniewski et al., 2012). As shown in **Figure 4**, sulfur metabolizing (including oxidation and reduction) genes were among the most abundant genes found in the metatranscriptome, and a significant difference (*p*-value < 0.001) in the expression profiles of sulfur metabolizing genes was observed between the chimney and the plume metatranscriptome. Therefore, the sulfur-metabolizing genes were highly abundant and expressed in this GB chimney sample, and displayed significantly higher expression pattern than those of a hydrothermal vent plume sample from Guaymas Basin.

### Carbon Metabolism

In this study, the complete WL pathway was identified in Archaeoglobales with high expression levels (**Supplementary Table S2**). The CBB cycle was not identified. The genes involved in the complete rTCA cycle were found to be actively present in both Deltaproteobacteria and Epsilonproteobacteria that dominated this chimney microbial community (**Table 3**). The key gene in the rTCA cycle, ATP-citrate lyase (*acl*), identified in this study to exhibit the highest expression was from Epsilonproteobacteria and exhibited the highest similarity to *Sulfurovum*, a novel sulfur-, nitrate-, and thiosulfate-reducing and strictly anaerobic chemolithoautotroph bacterium isolated from a deep-sea hydrothermal vent chimney at the Central Indian Ridge (Mino et al., 2014). In this study, the key enzyme for the utilization of acetate, acetyl-CoA synthetase (*acd/acs*), was found to be expressed and was assigned to sulfate-reducing bacteria (SRB; bin21 as shown in **Table 3**). In addition, the rTCA cycle and WL pathway were found to be the main pathways for carbon fixation by the dominant Bacteria and Archaea, respectively. This result suggests that, in combination with sulfur metabolism, autotrophic carbon fixation may play an important role in the survival and dominance of these species in the community. Moreover, as shown in **Supplementary Table S3**, genes involved in the flagellar assembly process were found to be actively present in Desulfovibrionales (bin21). The active role of the flagellar system in SRB may facilitate the movement toward electron donors and nutrients that occurs under the highly fluctuating conditions resulting from eruptions of hydrothermal vents. SRB have been reported to have the potential to anaerobically oxidize diverse hydrocarbons, such as alkanes, in Guaymas Basin sediments and chimney samples (Rueter et al., 1994). In this study, the activity and expression level of the presumably key gene in fumarate addition, a process through which alkanes are added to the double bond of fumarate based on the activity of alkylsuccinate synthase (*ass*), was checked. The *ass* genes were found to be highly active in this community, as determined through their expression level, and the most highly expressed hits were from *Desulfoglaeba alkanexedens* (Agrawal and Gieg, 2013), a typical sulfate-reducing and alkane-oxidizing bacterium (**Supplementary Table S4**). Moreover, the enzymes required for the degradation of a variety of organic compounds, such as hydrocarbons, fatty acids, chitins and proteins, have been detected in both the metagenome and metatranscriptome (**Supplementary Table S5**). Despite their important roles in carbon and global sulfur cycle, the energy metabolism of SRB remains poorly understood. After taxonomic assignment (see Materials and Methods), cyctochrome c (*cytC*), formate dehydrogenase (*fdh*), F-type ATPase (*atp*), NADH-quinone oxidoreductase (*nuo*), electron transport complex protein (*rnf*) and hydrogenases, such as Ni/Fe-hydrogenase I (*hyaAB*) and hydrogenase nickel incorporation and accessory protein (*hypA* and *hypB*), were found with expressions and assigned to SRB (**Supplementary Table S6**). The presence of hydrogenases and *fdh* may suggest that H_2_ or formate and play important roles in the flow of electrons during sulfate reduction. As shown above, the sulfur cycle in this community was particularly intensive and closely interacted with the carbon cycle, including carbon fixation and hydrocarbon degradation, to sustain the primary production in this ecosystem.

**Table 3:** Genes identified in the rTCA pathway in Delta- and Epsilonproteobacteria species.

Gene name	Abbrevations	Assigned taxonomy^∗^		FPKM
			
		Bin	BLAST	
Malate dehydrogenase	*mdh*	–	Bacteria	424.81
Fumarate hydratase subunit alpha	*fumA*	bin21	Desulfovibrionales	355.77
Fumarate hydratase subunit beta	*fumB*	–	Bacteria	135.08
Fumarate hydratase, class II	*fumC*	–	Bacteria	0.00
Fumarate reductase, flavoprotein subunit	*frdA*	–	Desulfovibrionales	1242.93
Fumarate reductase, iron–sulfur subunit	*frdB*	–	Epsilonproteobacteria	7.09
Succinyl-CoA synthetase	*sucC*		Bacteria	57.34
Succinyl-CoA synthetase alpha subunit	*sucD*	–	Desulfobacterales	724.00
2-Oxoglutarate ferredoxin oxidoreductase subunit alpha	*korA*	–	Deltaproteobacteria	65.56
2-Oxoglutarate ferredoxin oxidoreductase subunit beta	*korB*	–	Epsilonproteobacteria	232.91
2-Oxoglutarate ferredoxin oxidoreductase subunit delta	*korD*	–	–	-
2-Oxoglutarate ferredoxin oxidoreductase subunit gamma	*korC*	–	Bacteria	71.01
Isocitrate dehydrogenase	*icdA*	–	Bacteria	93.51
Isocitrate dehydrogenase (NAD+)	*IDH3*	–	Bacteria	0.00
2-Methylisocitrate dehydratase	*acnB*	–	Proteobacteria	20.34
Aconitate hydratase	*acnA*	bin21	Desulfovibrionales	69.50
Aconitate hydratase 2	*acnB*	–	Proteobacteria	20.34
ATP-citrate lyase alpha-subunit	*aclA*	–	Epsilonproteobacteria	238.10
ATP-citrate lyase beta-subunit	*aclB*	–	–	-
Pyruvate ferredoxin oxidoreductase alpha subunit	*porA*	–	Epsilonproteobacteria	9.90
Pyruvate ferredoxin oxidoreductase beta subunit	*porB*	–	Bacteria	59.23
Pyruvate ferredoxin oxidoreductase delta subunit	*porD*	–	Bacteria	2.66
Pyruvate ferredoxin oxidoreductase gamma subunit	*porG*	–	Epsilonproteobacteria	44.25
ADP-forming acetyl-CoA synthetase	*acd*	bin21	–	136.79
Acetate kinase	*ack*	–	*Thermotogaceae*	22.44
Phosphate acetyltransferase	*pta*	–	*Caldisericaceae*	21.14
				424.81


*^∗^The taxonomy assignments were determined by two methods, as described in Section “Materials and Methods.” The binning index is explained in** Supplementary Table S1**. #*FPKM* is based on the maximal expression value of the annotated genes.*

### Nitrogen Metabolism

The key genes involved in the nitrogen metabolism were found, and some of these were found to be actively expressed (**Table 4**). Many Bacteria and Archaea have the potential to perform denitrification (Philippot, 2002), and numerous organic and inorganic compounds can be used as electron donors for denitrification. The genes involved in denitrification, including *nar* (nitrate reductase), *nap* (nitrate reductase), *nir* (nitrite reductase), *nor* (nitric oxide reductase) and *nosZ*, were found to be present in the metagenome. The *narG* gene was assigned to *Beggiatoa*, a nitrate-respiring and sulfide-oxidizing bacterium that has been found to dominate microbial mats in hydrothermal sediments in the Guaymas Basin (Winkel et al., 2014). *narJ* was found to be expressed in Alteromonadales, whereas *napA* and *napB* were found to be expressed in Epsilonproteobacteria. To summarize, a complete set of denitrification genes were found in the bacterial community of the chimney, though some of them were found at low expression levels (**Table 4**). Based on this observation, we propose that nitrogen denitrification present in this community is most likely mediated by Gammaproteobacteria and Epsilonproteobacteria, with electrons generated by the ORS pathway.

**Table 4:** Genes identified in the nitrogen metabolic pathway in the microbial community.

Gene name	Abbrevations	Assigned taxonomy^∗^		FPKM
			
		Bin	BLAST	
Nitrate reductase alpha subunit	*narG*	–	Thiotrichales	49.68
Nitrate reductase beta subunit	*narH*	–	–	2360.80
Nitrate reductase gamma subunit	*narI*	–	Bacteria	380.02
Nitrate reductase delta subunit	*narJ*	–	Alteromonadales	7.17
Periplasmic nitrate reductase NapA	*napA*	–	Epsilonproteobacteria	98.48
Cytochrome c-type protein NapB	*napB*	–	Epsilonproteobacteria	4.68
Nitrite reductase (NO-forming)	*nirK*	–	–	–
Nitrite reductase (NO-forming)	*nirS*	–	–	–
Nitric oxide reductase subunit B	*norB*	–	Epsilonproteobacteria	0.00
Nitric oxide reductase subunit C	*norC*	–	–	–
Nitrous-oxide reductase	*nosZ*	–	Proteobacteria	284.50


*^∗^The taxonomy assignments were determined by two methods, as described in Section “Materials and Methods.” The binning index is explained in **Supplementary Table S1**. #*FPKM* is based on the maximal expression value of the annotated genes.*

## Discussion

Since the discovery of the deep-sea hydrothermal ecosystem in 1977, it has been proposed that hydrogen sulfide-oxidizing chemoautotrophs may potentially sustain the primary production in these ecosystems (Kvenvolden et al., 1995), where hydrogen sulfide or sulfide is primarily supplied via the high temperatures of seawater-rock interactions in the subseafloor hydrothermal reaction zones (Jannasch and Mottl, 1985). The chemical and microbial oxidation and reduction reactions of sulfur compounds probably establish the overall sulfur metabolism in the ecosystem (Yamamoto and Takai, 2011). There is no doubt that the sulfur cycle is one of the most important microbial chemosynthetic pathways in the microbial habitats of hydrothermal vents, but few studies have attempted to characterize the process, particularly at the function and activity levels. To date, the mechanism through which a microbial community in hydrothermal fields can be fueled by sulfate metabolism remains unclear. In particular, metagenomic approaches have not been widely applied in studies of energy generation by the microbial sulfur cycle in hydrothermal systems. In this study, a combined metagenomic and metatranscriptomic study of a chimney in the Guaymas Basin provides insight into the complete sulfur cycle based on the results from not only the genomic but also the expression analysis, the combination of which has not been previously used for the analysis of a deep-sea hydrothermal vent chimney sample.

The accumulation of hydrogen sulfides at the outer chimney promoted the coupling of sulfide oxidation to the electron acceptors present in the nearby marine water, including oxygen and nitrate, as supported by the retrieval of the functional and expressed genes described herein (**Tables 2**–**4** and **Figure 3**). These findings suggest that the coupling between sulfur oxidation and denitrification may fuel some N-metabolizing microorganisms at the sulfide-enriched outer chimney. As proposed in this study, the microorganisms involved in this process were Epsilonproteobacteria as the sulfur-oxidizing bacteria, and Gammaproteobacteria and Epsilonproteobacteria as potential denitrifiers. The other sulfur-metabolizing group, namely sulfate-reducing prokaryotes, may use hydrogen and/or dissolved organic matter as electron donors, as hydrogenases and key genes for the degradation of organic compounds have been identified in this study (**Supplementary Tables S5** and **S6**).

Carbon fixation pathways other than the Calvin–Benson–Bassham (CBB) cycle have been found to exhibit a notable contribution to carbon fixation, mostly at deep-sea hydrothermal vents (Campbell and Cary, 2004). The rTCA cycle was found to be highly expressed in the dominant Delta- and Epsilonproteobacteria. The key enzyme for the utilization of acetate was also identified to be expressed in this study (**Table 3**). Generally, the rTCA cycle appears to be dominant in habitats with a temperature ranging from 20 to 90°C, whereas the CBB cycle and the Wood-Ljungdahl (WL) pathway may be the principal pathways at temperatures lower than 20°C and greater than 90°C, respectively (Hugler and Sievert, 2011). In the present sample, the CBB cycle was not found present, which is consistent with the fact that this sample was collected from a high-temperature condition (He et al., 2013). In addition, the enzymes for the degradation of a variety of organic compounds, such as hydrocarbons, fatty acids, chitins and proteins, have been detected at both DNA and RNA level (**Supplementary Table S5**). Together, all of these organic compounds may be the carbon source for this microbial community.

In this scenario, both autotrophic and heterotrophic SRB could inhabit the inner chimney (**Figure 3**), where sulfate reduction is coupled to carbon fixation and hydrocarbon oxidation. Based on the expression levels of key genes in rTCA (**Table 3**) and alkane degradation (**Supplementary Table S4**), hydrocarbon degradation might contribute substantially to the linking of S and C cycle at inner layer chimney. In another word, heterotrophic SRB, commonly found at vent systems, may be the major player in coordinating and influencing the S and C cycle. Compared the expression of key genes in sulfur metabolizing and the rest processes (**Figure 4**), the reduced sulfur would be quickly and intensively oxidized to fuel the community, where sulfate-reducing microbes were found dominated. The composition of the sulfate-reducing community was determined by the way that microbes perform carbon metabolism. In our sample, heterotrophic SRB was found prevalent with their capabilities in hydrocarbon degradation. This finding may improve our understanding on the structure, function, and interaction within microbial community in hydrothermal vent.

Meta-omics based approaches have the advantages in studying the entire microbial community without pure cultures or prior knowledge on the sample. Functional omics approaches, such as transcriptome and proteome, could further confirm the metabolic potential at the active level. More efforts will be spent on quantification and comparison of these function omics datasets. Together with *in situ* carbon stable isotope measurement, and lipid type and diversity analysis, the activity, rate and interaction of key process in a given environmental condition could be accessed and estimated.

## Materials and Methods

### Sample Collection and Processing

The sample 4558-6 under investigation was collected from the outer layer of a black-smoker chimney in the Guaymas Basin and was previously described through a metagenome-based study (He et al., 2013). The sample was fixed with RNAlater (Sigma-Aldrich, Munich, Germany) and stored at -80°C prior to DNA and RNA extraction. DNA isolation was conducted as described previously (Wang et al., 2013). Metagenome pyrosequencing was performed using a 454 Life Sciences GS FLX system with a practical limit of 400 bp. RNA was isolated with a RNA isolation kit (Omega Bio-Tek, Doraville, GA, USA) following the user’s manual provided by the manufacturer. RNA samples were treated with DNAse (Thermo) for 45 min at 37°C, and then used as a template for PCR to detect undigested DNA. The mRNA fraction was enriched through the enzymatic digestion of rRNA molecules (mRNA-ONLY Prokaryotic mRNA Isolation kit, Epicentre Biotechnologies, Madison, WI, USA) followed by the subtractive hybridization of rRNA with capture oligonucleotides (Ambion MICROBExpress kit, Life Technologies, Gaithersburg, MD, USA). The mRNA isolates were first amplified (MessageAmp II-Bacteria kit, Ambion, Life Technologies) and then reversely transcribed into complementary DNA. Afterward, the cDNA was directly sequenced using the Illumina (BGI-Shenzhen, China) Hiseq2000 platform (2^∗^90 bp pair-end) for metatranscriptome analysis.

### Metagenome Assembly and Annotation

The reads obtained through metagenome sequencing were assembled and annotated as previously described (He et al., 2013). Briefly, low quality sequencing reads were trimmed in Geneious 6.04 (Biomatters Ltd.) and technical replicates were removed with cd-hit (at 96% sequence identity; Fu et al., 2012). After removing short reads (<100 bp), the remaining reads were assembled with Velvet (Zerbino and Birney, 2008). Coding regions of the metagenomic assembly were predicted using FragGeneScan (Rho et al., 2010) and then BLASTed (Altschul et al., 1997; 1e^-5^) against an NCBI non-redundant (NR) protein database. The 16S rRNA genes were picked using Sortmerna and BLASTed against GreenGene database (e-value < 1e^-5^) respectively. For functional annotation, sequences with matches to the COG (Tatusov et al., 2003), Pfam (Finn et al., 2014), and KEGG (Ogata et al., 1999) databases were retrieved to establish the functional categories and reconstruct the metabolic pathways. The genes of interest, such as transposases, were subjected to manual checkup, and spurious annotations (putative, like-, similar to) were excluded from further analysis.

### Taxonomic Assignment

Two different methods were applied to assess the taxonomic information. First, the assembled metagenomic sequences was binned using the tetranucleotide frequencies in emergent self-organizing maps (ESOMs; Dick et al., 2009) with a window size of 8 kbp, a sliding window size of 4 kbp, and the minimum fragment size of 2 kbp. Complete genomic sequences of 20 species were used as references (designated as bin1–20), these microorganism were listed as following: *Acinetobacter pittii* ANC 4052, *Alteromonas macleodii* str. ‘Deep ecotype,’ *Candidatus Pelagibacter ubique* HTCC1062, uncultured marine crenarchaeote E37-7F, Marine group II euryarchaeote SCGC AAA288-C18, Marine Group II euryarchaeote SCGC AB-629-J06, uncultured marine group II euryarchaeote (marine metagenome), Marine Group III euryarchaeote SCGC AAA007-O11, Marine Group III euryarchaeote SCGC AAA288-E19, *Marinobacter nanhaiticus* D15-8W, *Methylobacter tundripaludum* SV96, *Methylophaga aminisulfidivorans* MP, *Methylotenera mobilis* JLW8, *Nitrosopumilus maritimus* SCM1, *Candidatus Nitrospira defluvii*, *Planctopirus limnophila* DSM 3776, *Pseudomonas denitrificans* ATCC 13867, *Candidatus Ruthia magnifica* str. Cm (Calyptogena magnifica), SAR324 cluster bacterium SCGC AAA240-J09 and SAR86 cluster bacterium SAR86E. After binning, the completeness and taxonomic classification of the genomes within bins were then estimated by counting and BLASTing universal single-copy genes as previously described (Rinke et al., 2013). Alternatively, each predicted sequence feature in the metagenome and metatranscriptome was assigned to a certain taxon if at least 75% of the BLAST hits of this query were from that specific taxon. A BLAST search of all of the reads against the non-redundant protein database in NR was performed. All of the hits obtained from the BLAST searches were retained, and their taxonomic affiliations were determined using MEGAN (Huson et al., 2007) with bit-score values of 100. The taxonomic compositions of each predicted gene feature was then visualized using MEGAN.

### Metatranscriptome Mapping and Transcript Quantification

The raw shotgun sequencing metatranscriptomic reads obtained by Illumina pair-end sequencing were dereplicated (100% identity over 100% lengths) and trimmed using sickle^1^. The dereplicated, trimmed, and paired-end Illumina reads were then mapped to the metagenome using Bowtie (Langmead and Salzberg, 2012) with the default parameters. The unique mapped reads were selected, and FPKM (expected fragments per kilobase of transcript per million fragments mapped) was used to estimate the expression level of each gene using a script downloaded from GitHub^2^.

### Estimation of the Completeness of Genomic Bins

The complete genome sizes of the genomic bins were estimated based on an analysis of conserved single-copy genes (CSCGs) as described by Lloyd et al. (2013). In total, we were able to collect 162 and 139 universal CSCGs for the archaea and bacteria genomes, as in the previous study (Rinke et al., 2013). The ratios between the numbers of CSCGs present in the metagenome and the number of total CSCGs were then used to estimate the size of each genome bin.

### Comparative Analysis

The expression patterns of the sulfur-metabolizing genes in this metatranscriptome were compared to those in the metatranscriptome of a plum sample from Guaymas Basin (Lesniewski et al., 2012). Comparisons between two metatranscriptomes were conducted using the Mann–Whitney *U*-test. The gene expression profiles were compared between two samples using the normalized rank from 0 to 1 in each respective sample as the input. A difference was considered significant if the *p*-value was lower than 0.001.

### Construction of a Phylogenetic Tree

The predicted sequence features were checked across multiple annotation databases and then aligned with ClustalW (Larkin et al., 2007), and any gaps were removed manually. To construct functional gene phylogenies, the aligned sequences were analyzed by maximum likelihood-based FastTree (Price et al., 2010) using the Jones–Taylor–Thornton (JTT) with CAT approximation.

### Metabolic Pathway Identification

The gene products were searched for similarity against the KEGG database. A match was counted if the similarity search resulted in an expectation e-value below 1e^-5^. All of the occurring KO (KEGG Orthology) numbers were mapped against the KEGG pathway functional hierarchies and the COG database. For genes with multiple hits, only the genes with the highest expression value (FPKM) are displayed in the figures and tables and further discussed in the text.

### Data Availability

The metatranscriptome sequences are available on NCBI as SRX1008212. The assembled sequence was uploaded to IMG with a project ID Ga0072503.

## Conflict of Interest Statement

The authors declare that the research was conducted in the absence of any commercial or financial relationships that could be construed as a potential conflict of interest.

## Footnotes

1 https://github.com/najoshi/sickle

2 https://github.com/minillinim/sam2FPKG
