# Supplementary material for: Metagenome and Metatranscriptome Revealed a Highly Active and Intensive Sulfur Cycle in an Oil-Immersed Hydrothermal Chimney in Guaymas Basin
Source: Front Microbiol. 2015 Nov 10;6:1236. doi: 10.3389/fmicb.2015.01236 (PMC4639633; doi:10.3389/fmicb.2015.01236)
Supplement: Supplementary file 1 [file Data_Sheet_1.DOCX]

Fig. S1 Map of the binning results.

Fig. S2 Maximum likelihood-based phylogenetic tree of the *aprA* (A) and *dsrA* (B) genes. The bootstrap values are based on 1000 replicates, and the percentages are shown at the nodes. The genes identified in this study are highlighted with black dots. Numbers of genomes in each collapsed clade are displayed before the clade name.

**Fig. S1**

**
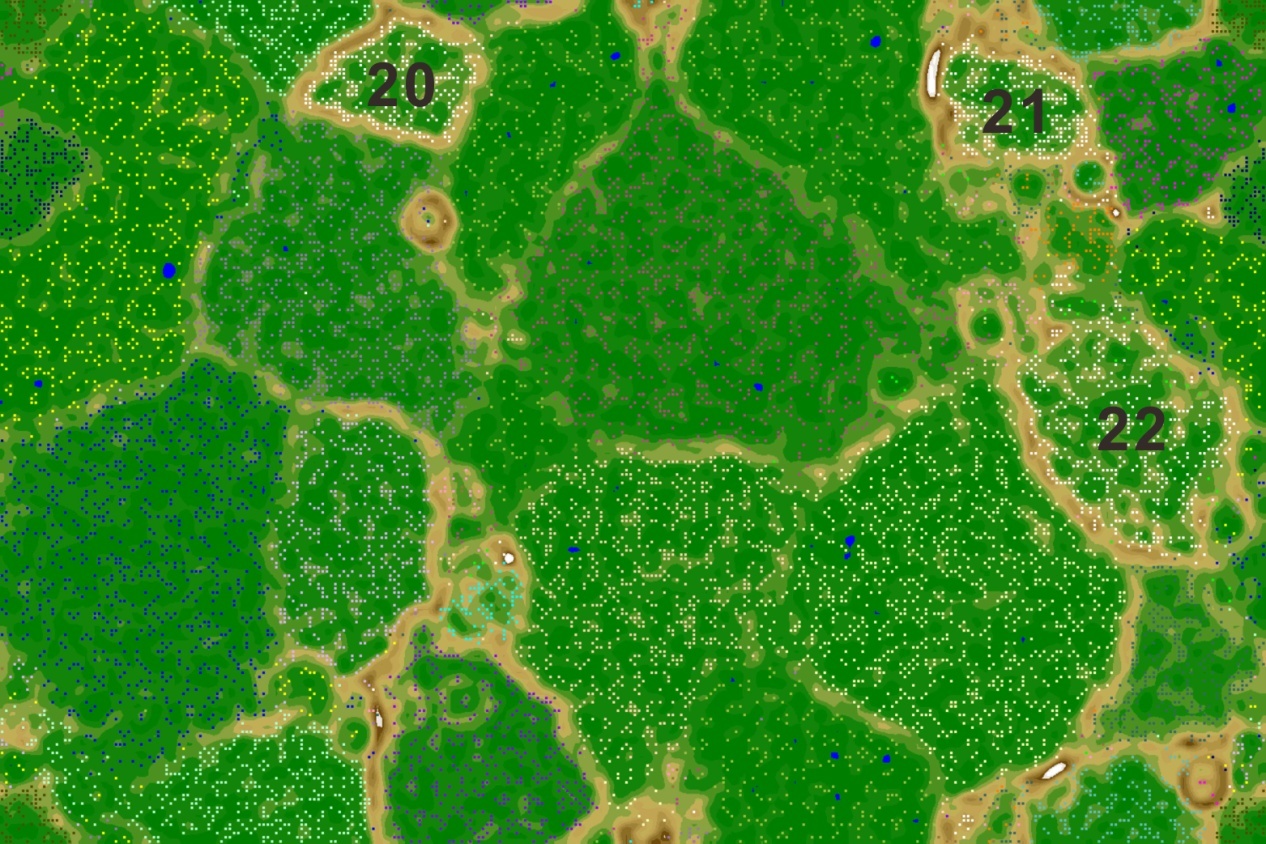
**

**Fig. S2A**

**
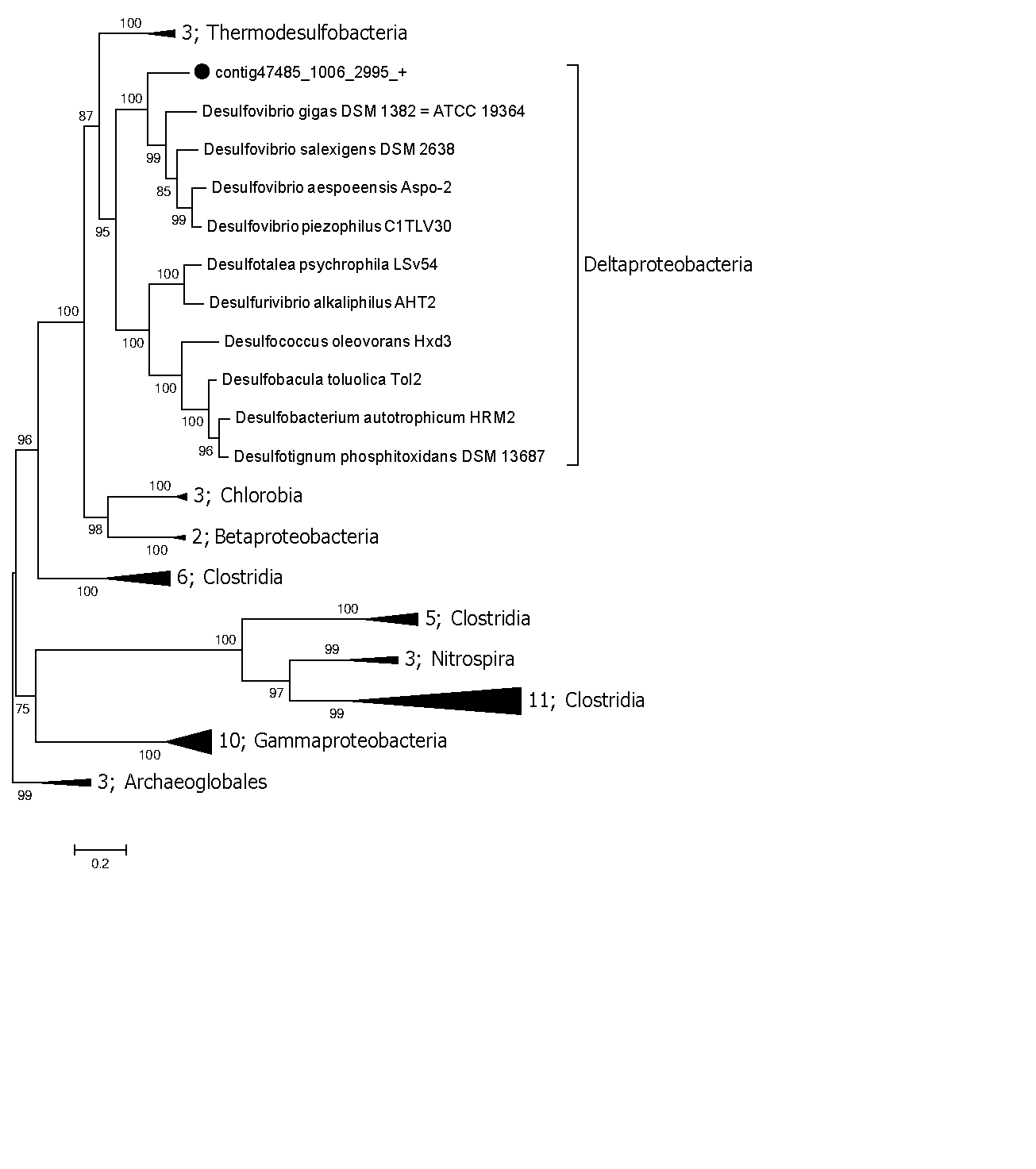
**

**Fig. S2B**

**
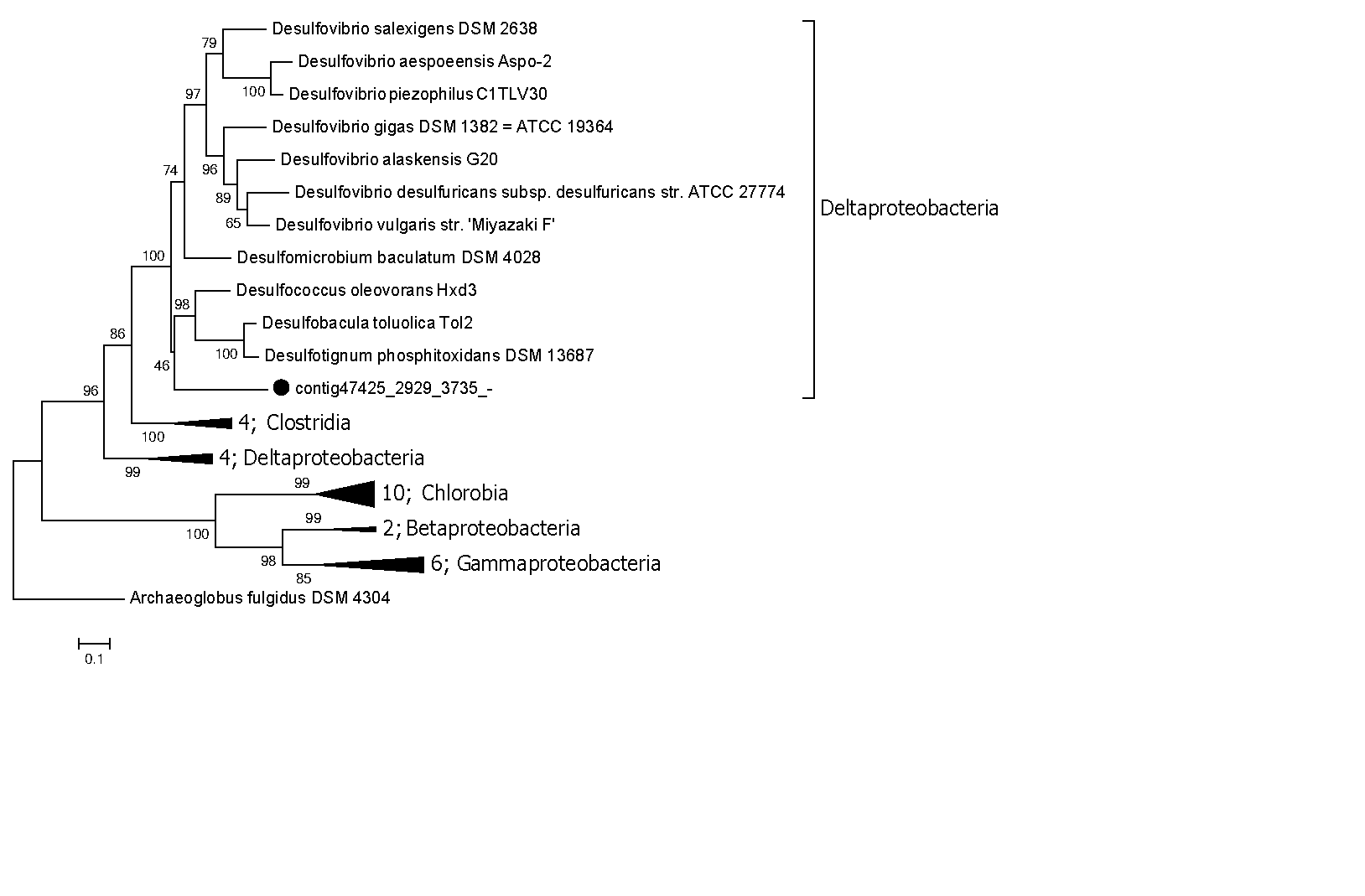
**

Table S1 General statistics of the identified genomic bins in the microbial community.

| Bin index | 20 | 21 | 22 |
| --- | --- | --- | --- |
| Bin size (Mb) | 0.88 | 1.1 | 2.05 |
| Number of contigs | 277 | 319 | 440 |
| Number of predicted genes | 486 | 782 | 1224 |
| No. of genes annotated to the Pfam database | 398 | 639 | 869 |
| No. of CSCGs* | 15 | 31 | 47 |
| Taxonomy of genomic bin | *Desulfobacteraceae* | Desulfovibrionales | *Archaeoglobus* |
| Expected complete genome size (Mb) | 9.504 | 4.932 | 6.063 |
| Achieved genome coverage (Pfam) | 9.3% | 22.3% | 33.8% |

The complete CSCG list is defined by Rinke et al. (Rinke et al., 2013). For the archaea genome, the total number of CSCGs is 162, whereas for bacteria, the total number is 139.

Table S2 Gene identified in the WL pathway.

| Gene name | Abbrev. | Assigned taxonomy* | | FPKM^#^ |
| --- | --- | --- | --- | --- |
|  |  | Bin | BLAST |  |
| Formylmethanofuran dehydrogenase subunit A | *fmdA* | bin22 | *Archaeoglobus* | 1241.20 |
| Formylmethanofuran dehydrogenase subunit B | *fmdB* | - | *Archaeoglobus* | 107.88 |
| Formylmethanofuran dehydrogenase subunit C | *fmdC* | - | - | - |
| Formylmethanofuran dehydrogenase subunit D | *fmdD* | - | *Archaeoglobus* | 379.71 |
| Formylmethanofuran tetrahydromethanopterin N-formyltransferase | *ftr* | bin22 | *Archaeoglobus* | 963.71 |
| Methenyltetrahydromethanopterin cyclohydrolase | *mch* | - | *Archaeoglobus* | 1571.43 |
| Methylenetetrahydromethanopterin dehydrogenase | *mtd* | bin22 | *Archaeoglobus* | 715.83 |
| 5,10-Methylenetetrahydromethanopterin reductase | *mer* | bin22 | *Archaeoglobus* | 917.93 |
| Acetyl-CoA decarbonylase/synthase alpha subunit | *cdhA* | bin22 | *Archaeoglobus* | 986.37 |
| Acetyl-CoA decarbonylase/synthase epsilon subunit | *cdhB* | - | *Archaeoglobus* | 385.96 |
| Acetyl-CoA decarbonylase/synthase beta subunit | *cdhC* | bin22 | *Archaeoglobus* | 554.45 |
| Acetyl-CoA decarbonylase/synthase delta subunit | *cdhD, acsD* | - | *Archaeoglobus* | 11.83 |
| Acetyl-CoA decarbonylase/synthase gamma subunit | *cdhE, acsC* | bin22 | *Archaeoglobus* | 361.76 |
| ADP-forming acetyl-CoA synthetase | *acd* | bin22 | - | 233.54 |

*The taxonomy assignments were determined by two methods, as described in Materials and Methods. The binning index is explained in Table S1. #FPKM is based on the maximal expression value of the annotated genes.

Table S3 Genes involved in the flagellar assembly process.

| Abbrev. | Gene name | Assigned taxonomy* | | FPKM^#^ |
| --- | --- | --- | --- | --- |
|  |  | Bin | BLAST |  |
| *FlhA* | Flagellar biosynthesis protein *FlhA* |  | Desulfovibrionales | 12.32 |
| *FlhB* | Flagellar biosynthesis protein *FlhB* |  | Bacteria | 54.05 |
| *FlhC* | Flagellar transcriptional activator |  | - | - |
| *FlhD* | Flagellar transcriptional activator *FlhD* |  | - | - |
| *FlgA* | Flagella basal body P-ring formation protein *FlgA* |  | Deltaproteobacteria | 8.28 |
| *FlgB* | Flagellar basal-body rod protein *FlgB* |  | *Helicobacteraceae* | 6.58 |
| *FlgC* | Flagellar basal-body rod protein *FlgC* |  | Bacteria | 4.34 |
| *FlgD* | Flagellar basal-body rod modification protein *FlgD* |  | Proteobacteria | 17.64 |
| *FlgE* | flagellar hook protein *FlgE* |  | Desulfovibrionales | 28.78 |
| *FlgF* | Flagellar basal-body rod protein *FlgF* |  | Thermotogae | 2.64 |
| *FlgG* | Flagellar basal-body rod protein *FlgG* |  | Desulfovibrionales | 41.88 |
| *FlgH* | Flagellar L-ring protein precursor *FlgH* |  | Desulfovibrionales | 14.11 |
| *FlgI* | Flagellar P-ring protein precursor *FlgI* |  | Desulfovibrionales | 24.37 |
| *FlgK* | flagellar hook-associated protein 1 *FlgK* |  | Desulfovibrionales | 27.52 |
| *FlgL* | flagellar hook-associated protein 3 *FlgL* |  | Desulfovibrionales | 62.58 |
| *FlgM* | Negative regulator of flagellin synthesis *FlgM* | bin21 | Bacteria | 9.93 |
| *FlgN* | Flagella synthesis protein *FlgN* |  | - | - |
| *FliC* | Flagellin |  |  | 181.58 |
| *FliD* | Flagellar hook-associated protein 2 *FliD* |  | Gammaproteobacteria | 76.47 |
| *FliE* | Flagellar hook-basal body complex protein *FliE* |  | Gammaproteobacteria | 3.92 |
| *FliF* | Flagellar M-ring protein *FliF* | bin21 | Desulfovibrionales | 25.79 |
| *FliG* | Flagellar motor switch protein *FliG* |  | Spirochaetales | 0.00 |
| *FliH* | Flagellar assembly protein *FliH* |  | - | - |
| *FliI* | Flagellum-specific ATP synthase |  | Bacteria | 16.32 |
| *FliJ* | Flagellar *FliJ* protein |  | Desulfovibrionales | 0.00 |
| *FliK* | Flagellar hook-length control protein *FliK* |  | Gammaproteobacteria | 0.00 |
| *FliM* | Flagellar motor switch protein *FliM* | bin21 | Desulfovibrionales | 59.06 |
| *FliN* | Flagellar motor switch protein *FliN* |  | *Helicobacteraceae* | 0.00 |
| *FliO* | Flagellar protein *FliO* |  | - | - |
| *FliP* | Flagellar biosynthetic protein *FliP* | bin21 | Desulfovibrionales | 115.24 |
| *FliQ* | Flagellar biosynthetic protein *FliQ* |  | Desulfovibrionales | 17.24 |
| *FliR* | Flagellar biosynthetic protein *FliR* |  | Deltaproteobacteria | 0.00 |
| *FliS* | Flagellar protein *FliS* |  | Desulfovibrionales | 406.74 |
| *FliT* | Flagellar protein *FliT* |  | - | - |
| *MotA* | Chemotaxis protein *MotA* |  | Desulfovibrionales | 69.01 |
| *MotB* | Chemotaxis protein *MotB* |  | Desulfovibrionales | 74.56 |

*The taxonomy assignments were determined by two methods, as described in Materials and Methods. The binning index is explained in Table S1. #FPKM is based on the maximal expression value of the annotated genes.

Table S4 The alkylsuccinate synthase (*ass*) genes in fumarate addition.

| Gene ID | E-value | Best Blast Hit Organism | FPKM^#^ |
| --- | --- | --- | --- |
| contig47456_1_630_- | 3.00E-114 | *Desulfoglaeba alkanexedens* ALDC | 3849.21 |
| contig46315_1_462_- | 3.00E-80 | *Desulfoglaeba alkanexedens* ALDC | 2166.67 |
| contig46066_1_408_+ | 2.00E-69 | *Desulfoglaeba alkanexedens* ALDC | 1838.24 |
| contig21221_1_235_- | 2.00E-22 | *Desulfoglaeba alkanexedens* ALDC | 1817.02 |
| contig45602_1_512_- | 2.00E-95 | *Desulfoglaeba alkanexedens* ALDC | 1423.83 |
| contig46307_1_218_- | 2.00E-17 | *Desulfoglaeba alkanexedens* ALDC | 1288.99 |
| contig45063_1_819_+ | 1.00E-157 | *Desulfoglaeba alkanexedens* ALDC | 1190.48 |
| contig42989_1_289_- | 4.00E-35 | *Sulfate-reducing bacterium* AK-01 | 1093.43 |
| contig46800_1_277_+ | 2.00E-23 | *Desulfoglaeba alkanexedens* ALDC | 794.22 |
| contig00513_1_275_- | 1.00E-05 | *Desulfoglaeba alkanexedens* ALDC | 730.91 |
| contig06644_1_346_- | 7.00E-48 | *Desulfoglaeba alkanexedens* ALDC | 450.87 |
| contig45077_1_179_+ | 1.00E-10 | *Desulfoglaeba alkanexedens* ALDC | 424.58 |
| contig48251_1_152_- | 5.00E-17 | *Desulfoglaeba alkanexedens* ALDC | 421.05 |
| contig48332_154_592_- | 2.00E-63 | *Desulfoglaeba alkanexedens* ALDC | 412.3 |
| contig22021_1_446_- | 2.00E-37 | *Sulfate-reducing bacterium* AK-01 | 289.24 |
| contig46024_1_323_+ | 6.00E-48 | *Desulfoglaeba alkanexedens* ALDC | 244.58 |
| contig46075_1_308_+ | 5.00E-46 | *Sulfate-reducing bacterium* AK-01 | 240.26 |
| contig48629_1_192_+ | 2.00E-29 | *Desulfoglaeba alkanexedens* ALDC | 93.75 |
| contig03908_1_237_- | 2.00E-35 | *Sulfate-reducing bacterium* AK-01 | 88.61 |
| contig22522_82_705_- | 5.00E-07 | *Desulfoglaeba alkanexedens* ALDC | 30.45 |
| contig21985_1_239_+ | 4.00E-25 | *Aromatoleum sp.* OcN1 | 29.29 |

*The taxonomy assignments were determined by two methods, as described in Materials and Methods. The binning index is explained in Table S1. #FPKM is based on the maximal expression value of the annotated genes.

Table S5 Key genes related to degradation of complex substrates.

| Substrate | Gene name | Abbrev. | Assigned taxonomy* | | FPKM^#^ |
| --- | --- | --- | --- | --- | --- |
|  |  |  | Bin | Blast |  |
| cellulose | cellulose | *celA* | - | Bacteria | 10.15 |
| chitin | chitinase | *chiA* | - | *Desulfococcus oleovorans* Hxd3 | 7.51 |
| starch | alpha-amylase | *amyA* | bin21 | Deltaproteobacteria | 15.57 |
| fatty acid | long-chain acyl-CoA synthetase | *fadD* | - | Bacteria | 498.13 |
| n-alkanes | alkylsuccinate synthase | *ass* | - | Proteobacteria | 3849.21 |
| ethylbenzene | ethylbenzene hydroxylase | *ebd* | bin20 | *Desulfococcus oleovorans* Hxd3 | 148.79 |
| toluene | benzylsuccinate synthase | *bss* | - | Firmicutes | 0 |

Table S6 Energy metabolism genes that are assigned to SRB.

| Abbrev. | Gene name | Assigned taxonomy* | | FPKM^#^ |
| --- | --- | --- | --- | --- |
|  |  | Bin | BLAST |  |
| *FdhA* | formate dehydrogenase subunit alpha | bin20 | - | 4.14 |
| *FdhD* | formate dehydrogenase accessory protein | - | *Desulfovibrio* | 37.67 |
| *etfA* | electron transfer flavoprotein alpha subunit | - | *Desulfococcus oleovorans* Hxd3 | 122.19 |
| *etfB* | electron transfer flavoprotein beta subunit | bin21 | *Desulfobacteraceae* | 548.53 |
| *rnfA* | electron transport complex protein *rnfA* | bin21 | *Desulfohalobium retbaense* DSM 5692 | 50.26 |
| *rnfB* | electron transport complex protein *rnfB* | bin20 | *Desulfococcus oleovorans* Hxd3 | 24.15 |
| *rnfB* | electron transport complex protein *rnfB* | bin21 | *Desulfohalobium retbaense* DSM 5692 | 189.14 |
| *rnfC* | electron transport complex protein *rnfC* | bin20 | *Desulfococcus oleovorans* Hxd3 | 30.66 |
| *rnfD* | electron transport complex protein *rnfD* | - | *Desulfococcus oleovorans* Hxd3 | 52.81 |
| *rnfE* | electron transport complex protein *rnfE* | bin21 | *Desulfohalobium retbaense* DSM 5692 | 34.60 |
| *rnfG* | electron transport complex protein *rnfG* | bin20 | *Desulfococcus oleovorans* Hxd3 | 47.14 |
| *nuoA* | NADH-quinone oxidoreductase subunit A | - | Desulfuromonadales | 2.92 |
| *nuoB* | NADH-quinone oxidoreductase subunit B | - | *Desulfobacca acetoxidans* DSM 11109 | 306.26 |
| *nuoD* | NADH-quinone oxidoreductase subunit D | bin21 | - | 287.30 |
| *nuoE* | NADH-quinone oxidoreductase subunit E | - | *Desulfobacula toluolica* Tol2 | 36.41 |
| *nuoF* | NADH-quinone oxidoreductase subunit F | - | *Desulfobacca acetoxidans* DSM 11109 | 40.96 |
| *nuoG* | NADH-quinone oxidoreductase subunit G | - | *Geobacter* | 0.00 |
| *nuoH* | NADH-quinone oxidoreductase subunit H | - | *Hippea maritima* DSM 10411 | 6.98 |
| *nuoM* | NADH-quinone oxidoreductase subunit M | - | *Desulfobacterium* | 0.00 |
| *nuoN* | NADH-quinone oxidoreductase subunit N | - | *Hippea maritima* DSM 10411 | 0.00 |
| *atpA* | H+-transporting ATPase subunit A | bin20 | *Desulfobacteraceae* | 50.33 |
| *atpA* | H+-transporting ATPase subunit A | bin21 | Desulfovibrionales | 550.21 |
| *atpB* | H+-transporting ATPase subunit B | bin21 | - | 228.70 |
| *atpB* | H+-transporting ATPase subunit B | bin20 | *Desulfobacteraceae* | 71.00 |
| *atpC* | H+-transporting ATPase subunit C | - | *Desulfococcus oleovorans* Hxd3 | 18.54 |
| *atpE* | H+-transporting ATPase subunit E | bin21 | Desulfovibrionales | 1559.21 |
| *atpG* | H+-transporting ATPase subunit G | - | *Desulfobacteraceae* | 3.90 |
| *hypA* | hydrogenase nickel incorporation protein | bin21 | *Desulfovibrio* | 71.23 |
| *hypB* | hydrogenase accessory protein | bin21 | Deltaproteobacteria | 23.15 |
| *hdrA* | heterodisulfide reductase subunit A | bin21 | Desulfovibrionales | 268.72 |
| *hdrB* | heterodisulfide reductase subunit B | - | *Desulfobacca acetoxidans* DSM 11109 | 0.00 |
| *hdrC* | heterodisulfide reductase subunit C | - | Desulfovibrionales | 65.36 |
| *mvhA* | F420-non-reducing hydrogenase large subunit | - | Desulfobacterales | 24.10 |
| *mvhD* | F420-non-reducing hydrogenase iron-sulfur subunit | bin20 | - | 0.00 |
| *hyaA* | Ni/Fe-hydrogenase I small subunit | - | *Desulfobacteraceae* | 0.00 |
| *hyaB* | Ni/Fe-hydrogenase I large subunit | - | *Desulfococcus oleovorans* Hxd3 | 11.19 |
| *cytC* | Cytochrome C assembly protein | - | *Desulfarculus baarsii* DSM 2075 | 13.61 |
| *CcdA* | Cytochrome C biogenesis protein *CcdA* | bin20 | *Desulfococcus oleovorans* Hxd3 | 27.12 |
| *ResB* | Cytochrome c biogenesis protein *ResB* | - | *Desulfococcus oleovorans* Hxd3 | 7.58 |

*The taxonomy assignments were determined by two methods, as described in Materials and Methods. The binning index is explained in Table S1. #FPKM is based on the maximal expression value of the annotated genes.
